# A 3D Porous Gelatin-Alginate-Based-IPN Acts as an Efficient Promoter of Chondrogenesis from Human Adipose-Derived Stem Cells

**DOI:** 10.1155/2015/252909

**Published:** 2015-05-27

**Authors:** Sorina Dinescu, Bianca Galateanu, Eugen Radu, Anca Hermenean, Adriana Lungu, Izabela Cristina Stancu, Dana Jianu, Tudorita Tumbar, Marieta Costache

**Affiliations:** ^1^Department of Biochemistry and Molecular Biology, University of Bucharest, 91-95 Splaiul Independentei, 050095 Bucharest, Romania; ^2^Institute of Life Sciences, Vasile Goldis Western University of Arad, 86 Rebreanu, 310414 Arad, Romania; ^3^Molecular Biology and Pathology Research Lab “Molimagex”, University Hospital Bucharest, 169 Splaiul Independentei, 050098 Bucharest, Romania; ^4^Advanced Polymer Materials Group, University Politehnica of Bucharest, 1-7 Gheorghe Polizu Street, District 1, 011061 Bucharest, Romania; ^5^Proestetica Medical S.R.L., 38-40 T. Stefan Street, 011658 Bucharest, Romania; ^6^International Society of Regenerative Medicine and Surgery, 38-40 T. Stefan Street, 011658 Bucharest, Romania; ^7^Department of Molecular Biology and Genetics, Cornell University, Ithaca, NY 14853, USA

## Abstract

Cartilage has limited regeneration potential. Thus, there is an imperative need to develop new strategies for cartilage tissue engineering (CTE) amenable for clinical use. Recent CTE approaches rely on optimal cell-scaffold interactions, which require a great deal of optimization. In this study we attempt to build a novel gelatin- (G-) alginate- (A-) polyacrylamide (PAA) 3D interpenetrating network (IPN) with superior performance in promoting chondrogenesis from human adipose-derived stem cells (hADSCs). We show that our G-A-PAA scaffold is capable of supporting hADSCs proliferation and survival, with no apparent cytotoxic effect. Moreover, we find that after exposure to prochondrogenic conditions a key transcription factor known to induce chondrogenesis, namely, Sox9, is highly expressed in our hADSCs/G-A-PAA bioconstruct, along with cartilage specific markers such as collagen type II, CEP68, and COMP extracellular matrix (ECM) components. These data suggest that our G-A-PAA structural properties and formulation might enable hADSCs conversion towards functional chondrocytes. We conclude that our novel G-A-PAA biomatrix is a good candidate for prospective *in vivo* CTE applications.

## 1. Introduction

Articular cartilage has a very limited capacity for intrinsic healing. Because the tissue is avascular, the injury does not induce the typical wound healing response, characterized by inflammation and invasion of undifferentiated mesenchymal cells [1]. Depending on the nature and severity of the cartilage lesion, therapies currently used in cartilage tissue engineering (CTE) can either be directed towards tissue repair and reconstruction or towards local tissue regeneration [2]. To date, strategies elaborated to solve cartilage defects remain ineffective.

The underlying principle of cell-based cartilage regeneration therapy is based on the ability of stem cells or cartilage precursor cells directly injected to the injury site to convert into functional chondrocytes able to synthesize cartilaginous extracellular matrix (ECM). Cell-based regenerative therapies appear to be more efficient than classical surgical methods, rapidly providing the patient with better quality of life. However, in the long term these regenerative strategies did not prove superior to surgical procedures. This highlights the crucial need to optimize the cell type, appropriate scaffold, prochondrogenic factors added in the microenvironment, and the interaction between these components.

One of the most difficult tasks in current cell-based cartilage regenerative therapies is to identify the most suitable matrix corresponding to each cartilage tissue defect. To develop stable and efficient strategies for CTE, several material design approaches have been investigated [3–5]. The substrate scaffold should correspond to certain criteria in order to be appropriate for CTE: (i) appropriate 3D structure in order to recreate the* in vivo* microenvironment; (ii) appropriate porosity to allow cell migration and diffusion of molecules, nutrients, and oxygen; (iii) biocompatibility; (iv) biodegradability; (v) matrix design to allow its insertion by mini-invasive procedures [6].

Cell types found to be useful in cartilage repair strategies are represented either by differentiated (chondrocytes) or undifferentiated cells (embryonic stem cells, mesenchymal stem cells, adipose-derived stem cells [7], synovium-derived stem cells, and periosteum-derived progenitor cells). Identification of the correct cell source is a key aspect for a cell-based cartilage reconstruction strategy, since these cells must exhibit high chondrogenic potential. From this point of view, each cell type, from stem cells to chondrocytes, presents certain advantages and disadvantages, leaving the most efficient cell source still debatable.

Mature chondrocytes represent possible candidates for cartilage repair due to their ability to produce type II collagen, sulfated glycosaminoglycans (GAG), and other molecules specific for the cartilaginous ECM [8]. In the articular cartilage, only 1–5% of the volume is occupied by chondrocytes, which are sparsely spread within the self-secreted ECM, made of collagens, proteoglycans, and other noncollagenous proteins [9]. Considering the above mentioned cell-ECM ratio, although each chondrocyte has a high activity [10], the overall metabolic rate of the cartilage tissue is low and, consequently, conducts to a poor self-regeneration potential.

In contrast to primary chondrocytes, mesenchymal stem cells (MSCs) are more abundant within certain tissues, making them easier to isolate. Main sources for MSC isolation are bone marrow (BM-MSCs) and adipose tissue, but MSC*-like* cells were also identified in other tissue types including synovium, periosteum, skeletal muscle, umbilical cord vein, or placenta [11]. These cells are all capable of chondrogenic differentiation in appropriate culture environments [12]. Human adipose-derived stem cells (hADSCs) could represent a viable source of mesenchymal*-like* stem cells for CTE applications, due to their easy harvest [13, 14], high chondrogenic potential [13, 15, 16], and wound healing properties [17–20]. Despite significant progress, obtaining from hADSCs functional chondrocytes capable of secreting type II collagen in tridimensional scaffolds remains challenging [21].

Current regenerative strategies in CTE aim to develop appropriate scaffold capable of supporting chondrogenesis. The aim of this study is to evaluate the* in vitro* biological performance of a novel 3D porous scaffold, in terms of biocompatibility and the ability to support hADSCs chondrogenic differentiation. Specifically, we developed a tricomponent system based on interpenetrating polymer networks (IPNs) of gelatin (G), alginate (A), and polyacrylamide (PAA). We studied the evolution of prochondrogenic induced hADSCs commitment towards functional chondrocytes inside this 3D porous scaffold, which proved to be biocompatible and to possess physical and chemical properties similar to cartilage. This work contributes to current efforts to address the challenges of using smart biomatrices to unleash the potential of stem cells for curative CTE.

## 2. Materials and Methods

### 2.1. Scaffold Synthesis

Gelatin B (G) from bovine skin (Sigma) was used as 20% (w/v) aqueous solution. Sodium alginate (SA) was used as 4% (w/v) aqueous solution. Acrylamide (AA) for electrophoresis, >99% (HPLC) and* N*,*N*′-methylenebis(acrylamide) (MBA) 99%, triethanolamine (TEA), ammonium persulphate (APS), glutaric aldehyde (GA) as aqueous solution 25%, and calcium chloride anhydrous (CaCl_2_) were purchased from Sigma and used without further purification.

IPNs based on G, calcium alginate (A), and polyacrylamide (PAA) were prepared using a three-step procedure reported elsewhere [22]. The three components were used in a weight ratio of 14 : 1 : 20 between G, SA, and AA, with a total solid content (T%) of 21%. Initially, cross linked PAA (molar ratio between MBA and AA was 1.8 : 100) was generated through network-forming radical polymerization of synthetic monomers in the presence of the natural polymers. Redox initiation based on APS (1% molar with respect to AA and MBA) and TEA (1/2 molar with respect to APS) was used to perform the polymerization reaction under mild conditions. Briefly, the polymerization stock solution was prepared based on a protocol detailed elsewhere [22], through the dissolution of AA, MBA, and the corresponding amount of initiator in distilled water, under stirring, at room temperature (RT). 1 mL of this solution was further mixed with 8 mL of gel solution and with 1 mL of SA solution, at 40°C. The resulting viscous solution was degassed using an ultrasound bath (Elma S 30H, Elmasonic) for 15 minutes at 40°C. The polymerization accelerator, TEA, was added under vigorous stirring. The network-forming polymerization of AA and MBA was allowed to occur in Petri dishes, for 24 hours, at 40°C. Semi-IPNs consisting in cross-linked PAA and uncross-linked G and SA were obtained. Then, the materials were cooled for 2 hours at 4°C, to allow physical gelation of G. G was cross-linked through immersion in GA 0.5% for 24 hours, at RT, followed by the cross-linking of SA in a 1% CaCl_2_ aqueous solution, for 24 hours. Calcium alginate (A) is thus formed. The resulting G-A-PAA hydrogel was further purified in distilled water at 40°C for four days. Gravimetric measurement was used to assess the success of the IPNs formation through gel fraction (GF) analysis. The experiment consists in the assessment of GF as ration between remaining gel (dried) after extensive extraction of the eventual soluble fraction in distilled water, at 40°C, with respect to the total mass of obtained G-A-PAA product (dried) following the synthesis:

(1) $$\begin{array}{c} \text{GF}=\frac{m_{\text{gel}}}{m_{0}}\times 100. \end{array}$$

Freeze-drying is becoming a common procedure to generate porous scaffolds from highly hydrated materials and therefore we used it to obtain porous G-A-PAA [23].

Control G-A and PAA hydrogels with the same T% (21%) were synthesized using similar procedures. GF values were estimated also for these samples. Porous scaffolds were generated through freeze-drying.

### 2.2. Morphological Assessment of Porosity through SEM

Morphological characterization of the scaffolds was obtained through Scanning Electron Microscopy (SEM) analysis of the gold-coated cross sections of the freeze-dried hydrogels. The study was performed using a QUANTA INSPECT F SEM device equipped with a field emission gun (FEG) with 1.2 nm resolution and with an X-ray energy dispersive spectrometer (EDS). Porous hydrogels were imaged prior to and after cell-seeding (7 days after seeding). The samples were fixed using 4% p-formaldehyde and subjected to freeze-drying procedure.

### 2.3. Cell Culture Model

The human subcutaneous abdominal white adipose tissue was obtained from overweight women undergoing elective liposuction. All the medical procedures were performed in compliance with the Helsinki Declaration, with the approval of Proestetica Medical Center Committee (reference number 112/23.10.2013). All subjects were in good health and provided their written consent before participating in the study. The lipoaspirates (LAs) were immediately processed by collagenase digestion and the obtained suspension was centrifuged at 2400 rpm for 10 min. Then, the pellet was resuspended in Dulbecco's modified Eagle's medium (DMEM, Sigma-Aldrich, Co), supplemented with 40% fetal bovine serum (FBS). During 24 h the adherent stem cells were allowed to attach to the culture surface, and then the culture medium was changed to DMEM containing 10% FBS. After reaching 3rd passage of culture, the specific mesenchymal stem cells surface markers panel, CD29^+^, CD44^+^, CD73^+^, CD90^+^, CD105^+^, CD14^−^, CD31^−^, and CD45^−^, was confirmed by flow cytometry.

### 2.4. Cell-Scaffold Bioconstruct Achievement

hADSCs in the 3rd passage were seeded on top of G-A, G-A-PAA, and PAA biomatrices at an initial density of 2.5 × 10^5^ cells/cm^2^ for biocompatibility studies and of 10^6^ cells/cm^2^ for chondrogenic differentiation assessment. The cell suspension was allowed to diffuse through the IPNs for 1 hour, in order for the cells to adhere to the inner layers of the biomaterial. The porous 3D biohybrids resulted after the scaffolds were put in contact with hADSCs. These are further referred to as hADSCs/G-A, hADSCs/G-A-PAA, and hADSCs/PAA bioconstructs.

For biocompatibility assessment, the biohybrids were incubated in standard conditions of cultivation in MesenPRO RS Medium for 7 days, with the medium refreshed every 2 days. Regarding the chondrogenic differentiation protocol, 24 hours after seeding the bioconstructs were exposed to prochondrogenic conditions for 28 days, using StemPro Chondrogenesis Differentiation Kit (Gibco, Life Technologies, Foster City, CA). hADSCs potential of differentiation was assessed at 3, 7, 14, and 28 days after induction. The time point when the systems were first exposed to the chondrogenic cocktail was considered T0.

### 2.5. Spectrophotometric Assays

#### 2.5.1. MTT Assay

hADSCs capacity to proliferate into G-A, G-A-PAA, and PAA biomatrices was quantitatively determined using MTT spectrophotometric assay at 24 h and 7 days after seeding. In this context, all cell-scaffold bioconstructs were incubated in 1 mg/mL MTT (Thiazolyl Blue Tetrazolium Bromide) solution (Sigma Aldrich Co., Steinheim, Germany) for 8 hours. The resulted formazan crystals were observed in contrast phase using a Nikon Eclipse TS 100 microscope (Nikon Instruments Europe, Amsterdam, Netherlands) and then subjected to solubilisation in isopropanol for 2 hours. The absorbance of the resulting solution was measured by spectrophotometry at 550 nm (Appliskan Thermo Scientific, Waltham, MA, USA). The optical densities obtained are proportional to cell viability.

#### 2.5.2. LDH Assay

The LDH assay is based on the quantification of the cytosolic lactate dehydrogenase enzyme released in the culture medium by the cells with damaged membrane.

The environmental cytotoxic potential of the G-A, G-A-PAA, and PAA materials on the hADSCs was evaluated using “*In vitro* toxicology assay kit lactate dehydrogenase based” (Sigma Aldrich Co, Steinheim, Germany) according to the manufacturer's protocol. Briefly, the culture media were harvested at 24 h and 7 days after seeding and they were mixed with the solutions provided in the kit. After 20 minutes of incubation at room temperature and darkness, the reaction was stopped with 1N hydrochloric acid (HCl). The LDH concentration was determined by measuring the optic density of the resulting solutions at 490 nm (Appliskan Thermo Scientific, Waltham, MA, USA).

#### 2.5.3. Quantitative ELISA

Cartilage oligomeric matrix protein (COMP) protein expression was assessed using Quantikine ELISA Human COMP (R&D Systems, Abingdon, UK), following manufacturer's instructions. COMP levels were determined from culture supernatants collected at T0, 3, 7, 14, and 28 days of culture in chondrogenic conditions and stored at −20°C. Final results of the immunoassay were determined by measuring the samples optical density at 450 nm and using a 540 nm wavelength correction. Additionally, a standard curve has been generated using the standard provided in the kit and following the assay procedure instructions.

### 2.6. Fluorescence Labeling Assays

#### 2.6.1. Live/Dead Assay

Live/Dead fluorescence microscopy assay was performed to evaluate hADSCs viability and proliferation within the 3D culture systems, using the Live/Dead Kit (Invitrogen, Life Technologies, Foster City, CA). This fluorescence-based kit combines calcein AM and ethidium bromide to yield two-color discrimination of the population of live cells from the dead-cell population.

Briefly, at 24 h and 7 days after seeding, hADSCs/G-A, hADSCs/G-A-PAA, and hADSCs/PAA bioconstructs were incubated with a staining solution prepared according to manufacturer's instructions, for 15 minutes in the dark. Next, the stained 3D cultures were analyzed by fluorescence microscopy using an Olympus IX71 inverted microscope and images were captured with Cell F Imaging Software (Olympus, Hamburg, Germany, 2008). 3D images were captured using confocal microscopy (Leica TCS-SP5 confocal scanner system).

#### 2.6.2. Immunofluorescence and Actin Filaments Fluorescent Labeling

The protein expression of actin and tubulin cytoskeleton proteins and of the nuclear transcription factor SRY (sex determining region Y) box 9 (Sox9), collagen type II, and the centrosomal protein of 68 kDa (CEP68) chondrogenic specific markers were studied by confocal fluorescence microscopy using a Carl Zeiss LSM710 laser scanning microscope, with Zeiss 20x and 40x 0.5NA objectives. hADSCs morphology and cytoskeleton fibers distribution in contact with G-A and G-A-PAA scaffolds were studied at 48 h days after seeding, while chondrogenic specific markers were assessed at T0, 3, 7, 14, and 28 days post-hADSCs/G-A and hADSCs/G-A-PAA exposure to the prochondrogenic environment.

In order to fluorescently label the target proteins, both hADSCs/G-A and hADSCs/G-A-PAA constructs were fixed with 4% PFA for 8 h and permeabilized with 2% BSA/0.1% Triton X-100 solution at 4°C. Next, the constructs were incubated 4 h at 37°C with Alexa Fluor 488 Phalloidin (Molecular Probes, Life Technologies, Foster City, CA) for actin labeling and overnight, at 4°C with mouse clonal anti-Sox9 (Abcam, Cambridge, UK), goat polyclonal anti-CEP68, and goat polyclonal anti-Col2a1 (Santa-Cruz Biotechnology, Heidelberg, Germany) antibodies. The bioconstructs were further incubated in TRITC conjugated goat anti-mouse and FITC conjugated rabbit anti-goat secondary antibodies solutions for 1 h (Santa-Cruz Biotechnology, Heidelberg, Germany). After cell nuclei were stained with DAPI for 30 min, the resulting labeled constructs were inspected in confocal fluorescence microscopy. Carl Zeiss Zen 2010 software version 6.0 was used for image acquisition and analysis. The 405, 488, and 543 nm laser lines were used for excitation and fluorescence emission was detected at 490–515 nm for DAPI, 520–550 nm for FITC, and 600–680 nm for TRITC. The confocal aperture used corresponded to a back-projected size of 1 Airy unit. Some of the images were acquired as z-stacks, and a maximal projection algorithm was used for 3D reconstruction.

### 2.7. qPCR Quantification

hADSCs/G-A and hADSCs/G-A-PAA bioconstructs were cut into fragments and the total RNA was isolated using TRIzol Reagent (Invitrogen, Foster City, CA, USA) in accordance with the manufacturer's instructions. After the isolated total RNA was tested for purity and concentration on NanoDrop spectrophotometer (Shimadzu, Duisburg, Germany) and for integrity on the BioAnalyzer 2100 (Agilent Technologies, Waldbronn, Germany), one microgram of total cellular RNA was reverse-transcribed to corresponding cDNA using iScript cDNA Synthesis kit (BioRad, Hercules, CA, USA) in a reaction volume of 20 *μ*L. Real-Time RT-PCR was performed on a LightCycler 2.0 carrousel-based system using LightCycler Fast Start DNA Master SYBR Green I Kit (Roche, Mannheim, Germany). Thermal cycling conditions were 10 min at 95°C, followed by 45 cycles of 10 s at 95°C, 10 s at 56°C, and 20 s at 72°C. The sequences of the primers used for Sox9, collagen type II, CEP68, and COMP genes detection are presented in Table 1 and were purchased from Life Technologies. The mRNA expression levels of target genes were normalized to the levels of TATA binding protein (TBP) and Tyrosine 3-monooxygenase/tryptophan 5-monooxygenase activation protein (YWHAZ), which were used as reference genes and were assessed in the same experimental conditions. Additionally, mRNA levels of the target chondrogenic markers were compared to the mRNA levels of the same markers from a culture of mature human chondrocytes, as a reference sample.

### 2.8. Statistical Analysis

The spectrophotometric and gene expression data were statistically analyzed using GraphPad Prism 3.03 Software, one-way ANOVA, and Bonferroni test, while comparison between chondrogenic markers mRNA levels of expression was performed using GenEx Enterprise 5.4.2 software. The experiments were performed with *n* = 3 biological replicates and each data set is presented as the average of three replicates (mean ± standard deviation).

## 3. Results

### 3.1. IPN Formation

To evaluate the success of the IPNs formation, GF values were calculated based on gravimetric measurements and using (1). The results indicated high GF values ranging from 95 ± 6% for G-A to 98 ± 3% for PAA and 98 ± 2% for G-A-PAA hydrogels. This confirms the success of the network formation through the described synthesis procedure.

### 3.2. Capacity to Generate Porous Structures

**Figure 1:**
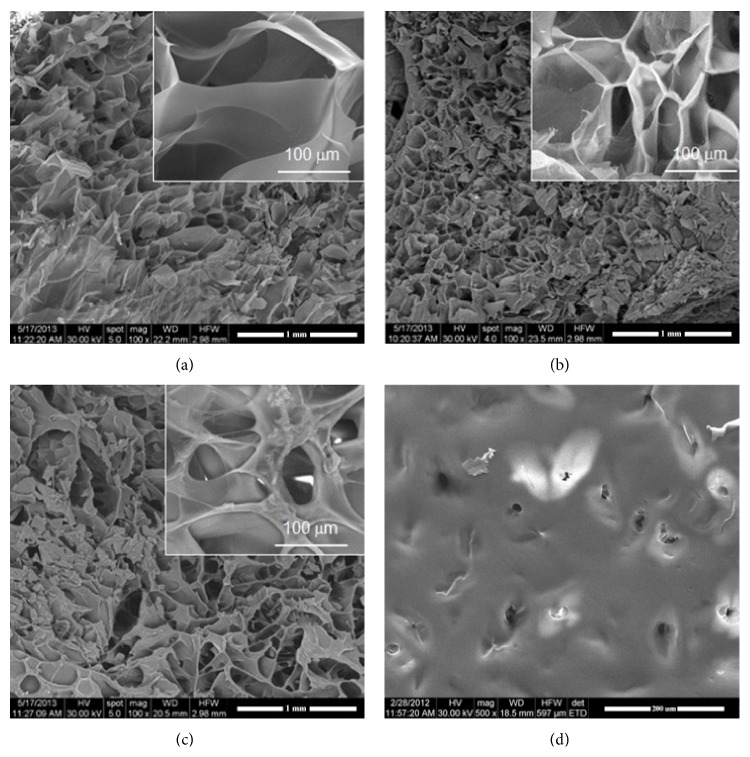
SEM micrographs showing the porosity of the unseeded scaffolds as visible through cross sections: (a) G-A-PAA, (b) G-A, (c) PAA, and (d) surface of PAA negative control.

Freeze-drying of the synthetized hydrogels generated porous materials with foam-like appearance. Morphological information on the pore shapes, homogeneity, sizes, and interconnection is given in Figure 1, as obtained from SEM investigation of the cross sections.

G-A IPN (Figure 1(b)) presents numerous interconnected pores with irregular still ovoidal shape and dimensions of approximately 100 *μ*m × 40 *μ*m. PAA (Figure 1(c)) also presents irregular pores with a different morphology, with thicker separation walls and larger dimensional distribution. Extremely interesting is that the studied G-A-PAA IPN (Figure 1(a)) does not combine the morphologies of the two control samples but has a totally novel architecture, with larger pores (approximately 200 *μ*m × 100 *μ*m), interconnected and with extremely thin and smooth separation walls.

### 3.3. Biocompatibility Assessment of Novel Porous Biomatrices in Contact with hADSCs

The biocompatibility of G-A-PAA novel IPN was tested considering G-A as reference scaffold and PAA as negative control, respectively. In this context, we evaluated the matrices' potential to support cellular viability and proliferation by Live/Dead and MTT assays, as well as their eventual cytotoxic effect using LDH test. Additionally, hADSCs distribution, morphology, and cytoskeleton organization inside the porous scaffolds were highlighted using SEM and confocal fluorescence microscopy.

#### 3.3.1. hADSCs Distribution inside the Porous Biomatrices

**Figure 2:**
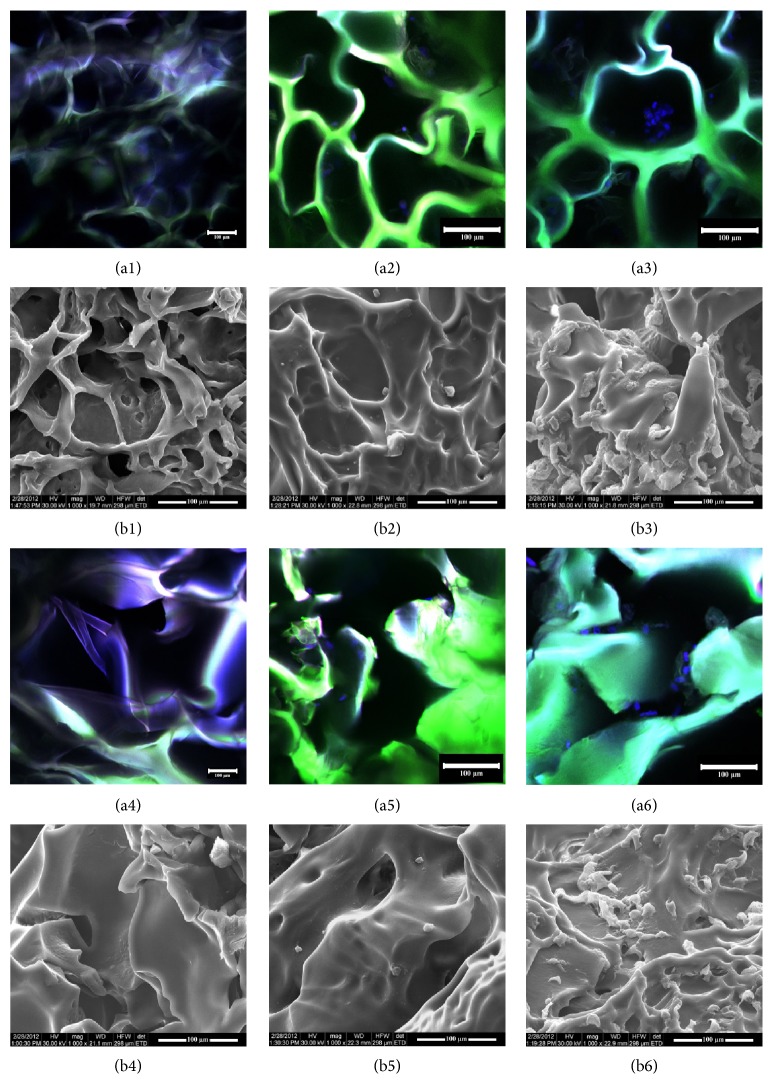
Confocal fluorescence microscopy (a) and SEM micrographs (b) showing G-A-PAA scaffold morphology: 1, unseeded; 2, at 24 h after seeding; 3, at 7 days after seeding; G-A scaffold morphology: 4, unseeded; 5, at 24 h after seeding; 6, at 7 days after seeding. Confocal microscopy images show the autofluorescence of the matrix (green) and DAPI-stained nuclei (blue).

SEM micrographs together with confocal fluorescence microscopy were performed to confirm cell distribution, shape, and proliferation trend. SEM analysis revealed the distribution of the cells within the porous hydrogels and the materials behavior in plain culture medium during one week of incubation in standard conditions of culture. Accordingly, as shown in Figure 2, after 7 days of culture, the biomaterials displayed an interconnected porous pattern and hADSCs populated deep layers of the scaffolds. Fluorescence confocal microscopy was performed at 2 and 7 days after seeding of the hADSCs in the G-A-PAA and G-A scaffolds after DAPI staining of nuclei. Accordingly, hADSCs proved to populate the pores of the scaffolds after 2 days of culture and continued to expand.

Furthermore, at 24 h of culture, hADSCs seeded in both G-A-PAA and G-A scaffolds displayed a spherical-*like* morphology (Figure 2 (a2)/(b2) and Figure 2 (a5)/(b5)), while after one week of incubation they adopted a characteristic spindle*-like* shape (Figure 2 (a3)/(b3) and Figure 2 (a6)/(b6)), suggesting their possible adhesion to the materials.

#### 3.3.2. hADSCs Morphology and Cytoskeleton Organization inside G-A-PAA and G-A IPNs

**Figure 3:**
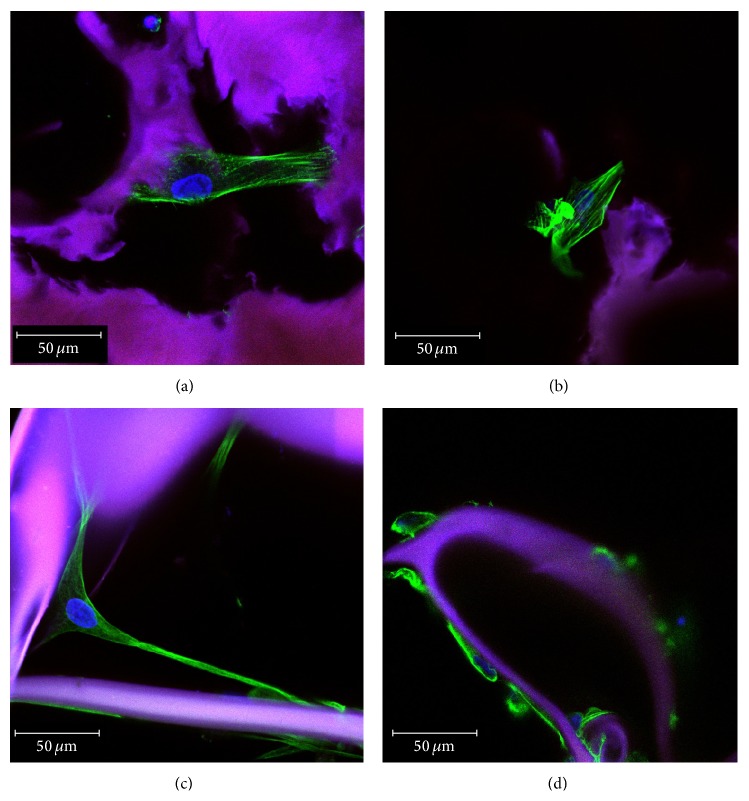
Confocal fluorescence microscopy micrographs of hADSCs actin filaments and tubulin network in hADSCs/G-A-PAA biohybrid ((a), (c)) and in hADSCs/G-A control ((b), (d)). Matrix autofluorescence is seen in purple; DAPI-stained nuclei are blue.

hADSCs morphology and the ability to interact with the substrate material in terms of adhesion and cytoskeleton development were carefully investigated once the hADSCs/G-A-PAA and hADSCs/G-A biohybrids were obtained. 48 h after seeding, hADSCs from both constructs displayed long and distinctive actin filaments surrounding the nuclei, which clearly determined cell overall morphology (Figures 3(a) and 3(b)). Consequently, hADSCs ability to dynamically form microfilaments may be considered part of the physiological shape modeling process in response to the stimulus represented by the substrate. The actin cytoskeleton underlies the cell adhesion process, which is highly important for further tissue formation.

The actin cytoskeleton is closely related to the microtubule network, regulating cell motility and maintenance of cell shape. Tubulin distribution (Figures 3(c) and 3(d)) inside the hADSCs seeded/grown/cultured in the scaffolds also revealed their spindle-*like* shape and attachment to the substrate.

#### 3.3.3. Live/Dead Fluorescence Microscopy Assay

**Figure 4:**
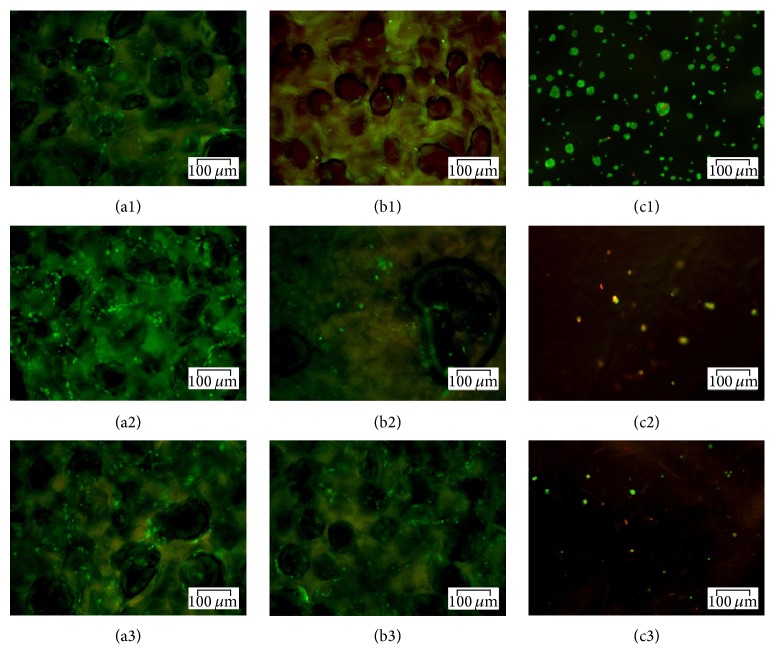
Fluorescence microscopy micrographs revealing live and dead cells in G-A-PAA (a), G-A (b), and PAA (c) matrices at 24 h (1), 5 days (2), and 7 days (3) of culture.

**Figure 5:**
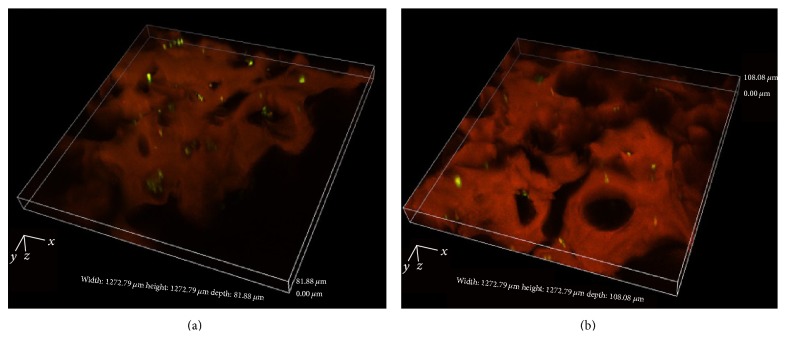
3D laser scanning reconstruction of Live/Dead stained (a) hADSCs/G-A-PAA bioconstruct and (b) hADSCs/G-A bioconstruct. Viable (green) and dead (red) cells are seen embedded in the autofluorescent matrix (orange).

In order to examine cell survival inside the tested biomaterials, the viability of hADSCs was evaluated at 24 h and 7 days after seeding by fluorescence (Figure 4) and confocal fluorescence (Figure 5) microscopy, based on the simultaneous staining of live (green labeled) and dead (red labeled) cells.

At 24 h after seeding bright green labeled cells were observed surrounding the G-A-PAA and G-A pores, while unattached spherical-shaped cells were displayed on top of PAA matrix. After one week of culture the number of green-labeled hADSCs inside both the sample and the control scaffold increased as compared to 24 h after seeding, confirming their proliferation. This observation could indicate that G-A-PAA and G-A sustain cellular viability and proliferation, offering a proper microenvironment to hADSCs. Regarding the negative control, the amount of cells seeded on top of the scaffold dramatically decreased after 7 days of culture.

Laser scanning of hADSCs/G-A-PAA and hADSCs/G-A bioconstructs confirmed previously presented SEM cell distribution data (Figure 2) and, in addition, showed that hADSCs inside pores were viable after one week of culture (Figure 5).

Furthermore, we observed an increased number of viable green labeled hADSCs inside G-A-PAA scaffold as compared to G-A control material, which populated in large groups the pores of the matrix.

#### 3.3.4. MTT

**Figure 6:**
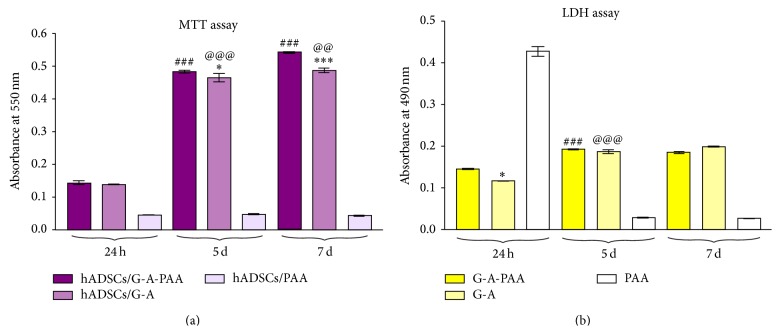
The quantification of (a) hADSCs proliferation rate in G-A-PAA, G-A, and PAA biomatrices, as revealed by MTT test and (b) the cytotoxic potential of G-A-PAA, G-A, and PAA biomatrices on hADSCs as revealed by LDH assay, at 24 h, 5 days, and 7 days. (^*^ *P* < 0.05 (hADSCs/G-A-PAA versus hADSCs/G-A bioconstruct); ^***^ *P* < 0.001 (hADSCs/G-A-PAA versus hADSCs/G-A bioconstruct); ^###^ *P* < 0.001 (hADSCs/G-A-PAA: 5 days versus 24 h days and 7 days versus 5 days); ^@@^ *P* < 0.01 (hADSCs/G-A: 7 days versus 5 days); ^@@@^ *P* < 0.001 (hADSCs/G-A: 5 days versus 24 h)).

To confirm hADSCs viability and the proliferation rate observed by fluorescence microscopy, MTT assay was employed as a more accurate approach. In this context, the hADSCs/G-A-PAA, hADSCs/G-A, and hADSCs/PAA bioconstructs were subjected to MTT spectrophotometric assay at 24 h and 5 and 7 days of culture (Figure 6(a)).

Our results showed that in PAA scaffold the level of the formazan concentration was almost undetectable during the entire experimental period. This suggested that hADSCs did not survive 24 h in contact with the negative control. In contrast, hADSCs inside G-A-PAA and G-A scaffolds proliferated during one week of experiment, with the same statistical significance increase (*P* < 0.001) between 24 h and 5 days after seeding. During the 5th and the 7th day of culture, the number of the metabolically active cells inside G-A-PAA matrix significantly increased (*P* < 0.001) when compared to hADSCs seeded in G-A control scaffold (*P* < 0.01).

No significant differences were observed in cellular viability between hADSCs/G-A-PAA and hADSCs/G-A scaffolds, as indicated by their absorbance values measured at 24 h. At 5 days of culture a slightly significant increase of cellular viability (*P* < 0.05) was detected for cells in G-A-PAA matrix as compared to the control biohybrid, while at 7 days after seeding the viability difference between these bioconstructs was found significantly higher (*P* < 0.001).

#### 3.3.5. LDH

The cytotoxic potential of G-A-PAA scaffold was evaluated by spectrophotometric quantification of the LDH enzyme release in the culture media by the embedded hADSCs. G-A and PAA matrices, preseeded with the same number of hADSCs, were used as reference and negative control, respectively (Figure 6(b)).

At 24 h after seeding, LDH levels detected in the culture medium harvested from the negative control biohybrid were found statistically significantly higher (*P* < 0.001) as compared to the results obtained for hADSCs/G-A-PAA and hADSCs/G-A biohybrids. This observation together with the detection of very low LDH levels in hADSCs/PAA at 5 and 7 days of culture conducted to the presumption that hADSCs did not survive in contact with PAA scaffold more than 24 h.

Although G-A-PAA scaffold displayed significantly higher (*P* < 0.05) levels of LDH as compared to the control biomatrix at 24 h, no differences were registered at 5 and 7 days of culture between these samples. Considering this profile, the higher levels of LDH released in the culture medium by hADSCs seeded in G-A-PAA at the beginning of the experiment could be determined by the unpolymerised AA residues, which were subsequently washed during the subcultivation procedures.

LDH concentration in hADSCs/G-A-PAA and hADSCs/G-A biohybrids was significantly higher at 5 days of culture (*P* < 0.001) than at 24 h, in accordance to cell proliferation rates. Consequently, the overall ratio between proliferation and cellular death is maintained throughout the experiment. At 7 days of culture no significant differences were noticed as compared to the previous experimental time point.

### 3.4. hADSCs* In Vitro* Chondrogenic Differentiation Assessment inside G-A-PAA Scaffold

The chondrogenic process in hADSCs/G-A-PAA and hADSCs/G-A bioconstructs was monitored for 28 days by the evaluation of early and late chondrogenic markers gene and protein levels of expression.

#### 3.4.1. qPCR Quantification of Sox9, CEP68, and COMP Chondrogenic Markers

**Figure 7:**
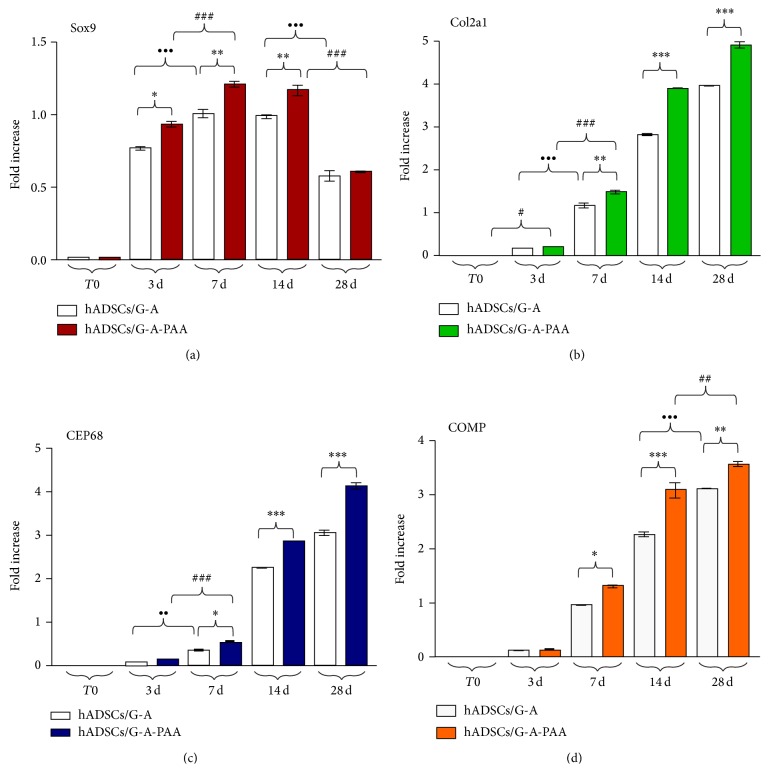
Gene expression profiles of (a) Sox9 (^*^ *P* < 0.05 (hADSCs/G-A-PAA versus hADSCs/G-A bioconstruct: 3 days); ^**^ *P* < 0.01 (hADSCs/G-A-PAA versus hADSCs/G-A bioconstruct: 7 days and 14 days); ^•••^ *P* < 0.001 (hADSCs/G-A: 7 days versus 3 days and 28 days versus 14 days); ^###^ *P* < 0.001 (hADSCs/G-A-PAA: 7 days versus 3 days and 28 days versus 14 days)), (b) Col2a1 (^**^ *P* < 0.01 (hADSCs/G-A-PAA versus hADSCs/G-A bioconstruct: 7 days); ^***^ *P* < 0.001 (hADSCs/G-A-PAA versus hADSCs/G-A bioconstruct: 14 days and 28 days); ^•••^ *P* < 0.001 (hADSCs/G-A: 7 days versus 3 days); ^#^ *P* < 0.05 (hADSCs/G-A-PAA: 3 days versus T0); ^###^ *P* < 0.001 (hADSCs/G-A-PAA: 7 days versus 3 days)), (c) CEP68 (^*^ *P* < 0.05 (hADSCs/G-A-PAA versus hADSCs/G-A bioconstruct: 7 days); ^***^ *P* < 0.001 (hADSCs/G-A-PAA versus hADSCs/G-A bioconstruct: 14 days and 28 days); ^••^ *P* < 0.01 (hADSCs/G-A: 7 days versus 3 days); ^###^ *P* < 0.001 (hADSCs/G-A-PAA: 7 days versus 3 days)), (d) COMP (^*^ *P* < 0.05 (hADSCs/G-A-PAA versus hADSCs/G-A bioconstruct: 7 days); ^**^ *P* < 0.01 (hADSCs/G-A-PAA versus hADSCs/G-A bioconstruct: 28 days); ^***^ *P* < 0.001 (hADSCs/G-A-PAA versus hADSCs/G-A bioconstruct: 14 days); ^•••^ *P* < 0.001 (hADSCs/G-A: 28 days versus 14 days); ^##^ *P* < 0.01 (hADSCs/G-A-PAA: 28 days versus 14 days)) as revealed by qPCR analysis and GraphPad Prism 3.0 data statistical analysis.

Molecular analysis of Sox9, collagen type II (Col2a1), CEP68, and COMP chondrogenic markers gene expression at 3, 7, 14, and 28 days after induction revealed a complex transcriptional and signaling pathway underlying the chondrogenic differentiation process (Figure 7). A sample harvested before the bioconstructs exposure to chondrogenic conditions was used as reference for gene expression analysis and will be further addressed as T0.

Sox9 was shown to play an essential role in cell differentiation to the chondrogenic pathway and in chondrocyte fate, thus being considered the master regulator and key inducer of the chondrogenic differentiation process. In our experiment, Sox9 mRNA high levels were detected at 3 days after induction in both hADSCs/G-A-PAA and hADSCs/G-A bioconstructs, suggesting its activation, as compared to the basal levels registered at T0 (Figure 7(a)). Interestingly, a statistically significantly higher level of Sox9 transcript (*P* < 0.05) was found in hADSCs/G-A-PAA than in the control biohybrid after 3 days of experiment. Furthermore, a significant upregulated Sox9 expression was found both in hADSCs/G-A-PAA bioconstruct (*P* < 0.001) and in control (*P* < 0.001) at 7 days postchondrogenic induction, as compared to the levels detected at 3 days. Together with this ascending expression profile found for the first week of experiment, corresponding to the initial activation of Sox9 chondrogenic inducer, Sox9 mRNA levels at 7 days were also found to be statistically significantly higher (*P* < 0.01) in hADSCs/G-A-PAA than in the control bioconstruct. Additionally, this difference was also present at 14 days of induced chondrogenesis, since Sox9 transcript levels were proved to remain constant between 7 and 14 days of experiment/during the second week of experiment. Notably, 28 days postchondrogenic induction, Sox9 mRNA levels registered a significant decrease (*P* < 0.001) in both biohybrids, while no important difference was found between hADSCs/G-A-PAA and hADSCs/G-A in terms of Sox9 pattern of expression.

In our particular conditions, Col2a1 gene expression was first detected at 3 days of induced chondrogenesis in hADSCs/G-A-PAA bioconstruct by comparison to Col2a1 levels at T0 (*P* < 0.05), suggesting that this ECM marker synthesis begins early during the chondrogenic differentiation process (Figure 7(b)). In contrast, collagen type II was statistically significant expressed for the first time in the control bioconstruct at 7 days after induction (*P* < 0.001). Furthermore, mRNA levels were found significantly higher in hADSCs/G-A-PAA than in hADSCs/G-A after one week of* in vitro* chondrogenesis (*P* < 0.01). Col2a1 transcript levels statistically increased (*P* < 0.001) between 7 and 14 days in both bioconstructs, also maintaining the difference in expression higher in hADSCs/G-A-PAA than in control (*P* < 0.001). This upregulated profile registered a constant and statistically significant increase (*P* < 0.001) in both bioconstructs, during 14–28 days interval. Overall, Col2a1 gene expression profile registered an increasing trend, suggesting collagen type II continuous synthesis, as proof of ECM accumulation.

CEP68 was detected in samples harvested from both bioconstructs starting with day 7 of prochondrogenic induction (Figure 7(c)), at statistically significantly higher levels (*P* < 0.05) in hADSCs/G-A-PAA than in control. Additionally, CEP68 mRNA levels of expression were found to be increased at 7 days, as compared to 3 days after induction both in hADSCs/G-A-PAA (*P* < 0.001) and in hADSCs/G-A (*P* < 0.01) systems. The general gene expression profile of CEP68 resembles Col2a1 trend, registering a gradual increase during the 28 days of* in vitro* chondrogenesis. Thus, CEP68 transcript levels significantly increased (*P* < 0.001) in both bioconstructs between 7 and 14 days, as well as in the last two weeks of experiment, while the statistically significant difference (*P* < 0.001) in CEP68 gene expression between hADSCs/G-A-PAA and hADSCs/G-A constructs was also maintained constant.

Similar to CEP68 ECM marker, COMP was first detected (*P* < 0.001) in hADSCs/G-A-PAA system at 7 days postchondrogenic induction, as compared to 3 days, with a gene expression significantly higher (*P* < 0.05) than the levels found in control (Figure 7(d)). COMP mRNA levels gradually increased in both constructs between 7 and 14 days (*P* < 0.001) of experiment, maintaining a statistically significant difference (*P* < 0.001) between COMP transcript levels in hADSCs/G-A-PAA and in hADSCs/G-A. Furthermore, COMP expression considerably increased (*P* < 0.001) in hADSCs/G-A bioconstruct after the second week, whereas simultaneously the levels in hADSCs/G-A-PAA registered a lower increase (*P* < 0.01).

#### 3.4.2. Qualitative Assessment of Sox9, Col2a1, and CEP68 Chondrogenic Markers

**Figure 8:**
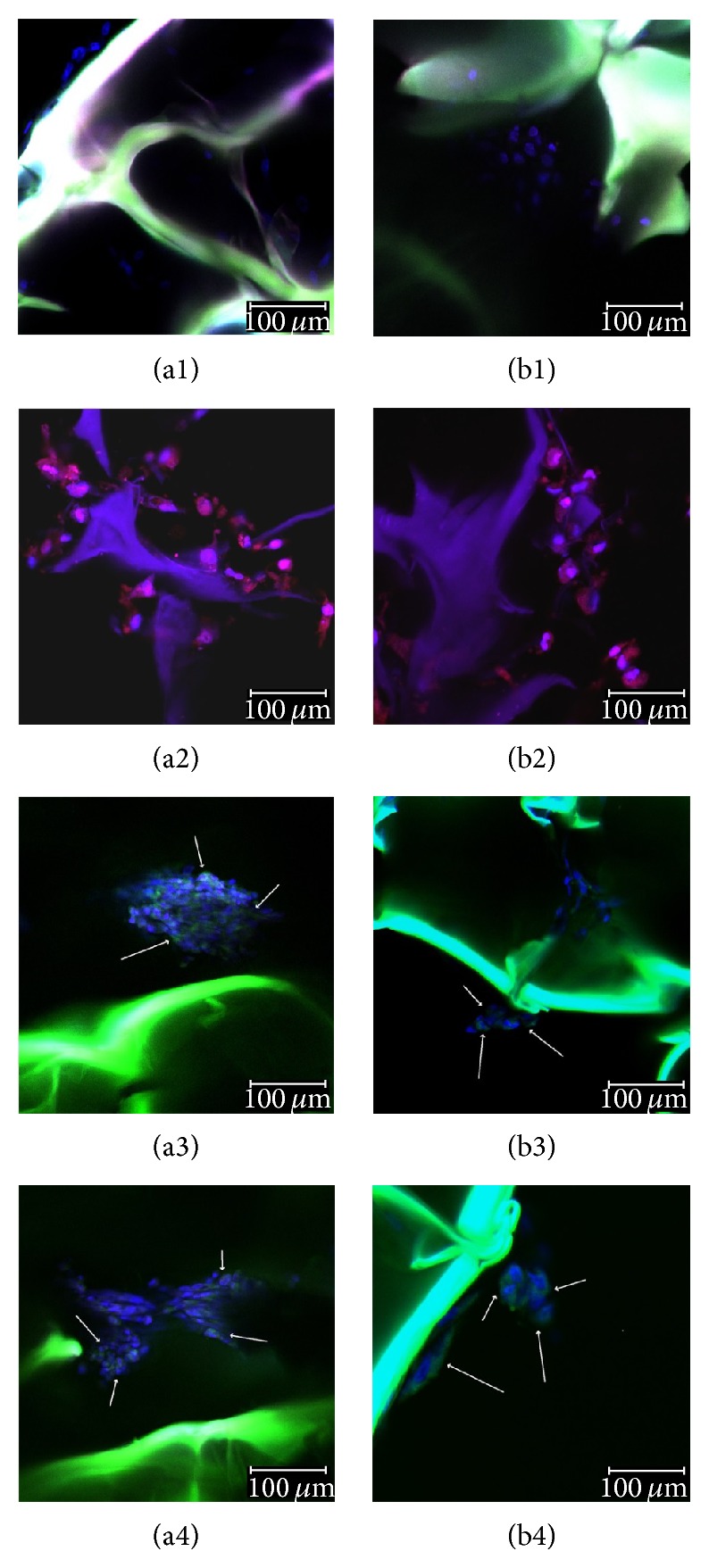
Confocal microscopy micrographs of cells inside (a) G-A-PAA and (b) G-A scaffolds during chondrogenesis: (1) 24 h after seeding, (2) Sox9 positive expression (red fluorescence) at 7 days postchondrogenic induction, (3) Col2a1 positive expression (green fluorescence) at 14 days postchondrogenic induction, and (4) CEP68 positive expression (green fluorescence) at 14 days postchondrogenic induction.

Sox9, Col2a1, and CEP68 protein expression was evaluated by confocal fluorescence microscopy and the captured micrographs displaying the earliest positive expression during the chondrogenic differentiation process are presented in Figure 8. A set of hADSCs/G-A-PAA and hADSCs/G-A bioconstructs unexposed to chondrogenic conditions were used as reference and proved the uncommitted state of the embedded hADSCs.

As shown in Figure 8(a2) and Figure 8(b2), Sox9 transcriptional factor was first expressed at protein level at 3 days postchondrogenic induction in both hADSCs/G-A-PAA and hADSCs/G-A bioconstructs. However, hADSCs in our sample were observed to condense in larger groups and to express more frequently Sox9, as compared to the cells in the control matrix. These observations are in accordance with the data obtained after Sox9 gene expression analysis (Figure 7(a)).

ECM synthesis is a specific process during chondrogenesis. The expression of Col2a1 and CEP68 ECM proteins is late chondrogenic marker, suggesting stem cell conversion towards mature chondrocytes. In our experimental conditions, both Col2a1 and CEP68 were first expressed after 7 days of chondrogenic induction, as also revealed by qPCR results (Figures 7(b) and 7(c)). In addition, using confocal fluorescence microscopy, net differences in cellular distribution inside G-A-PAA and G-A matrices were highlighted after one week of chondrogenesis. hADSCs inside G-A-PAA scaffold were found to be organized in large clusters and trapped in their self-secreted ECM, whereas differentiating hADSCs inside G-A control matrix barely assembled in small groups, as shown by the double staining of cell nuclei and ECM proteins (Figure 8(a3), (a4) and Figure 8(b3), (b4)).

#### 3.4.3. Quantitative Detection of COMP Protein Expression

**Figure 9:**
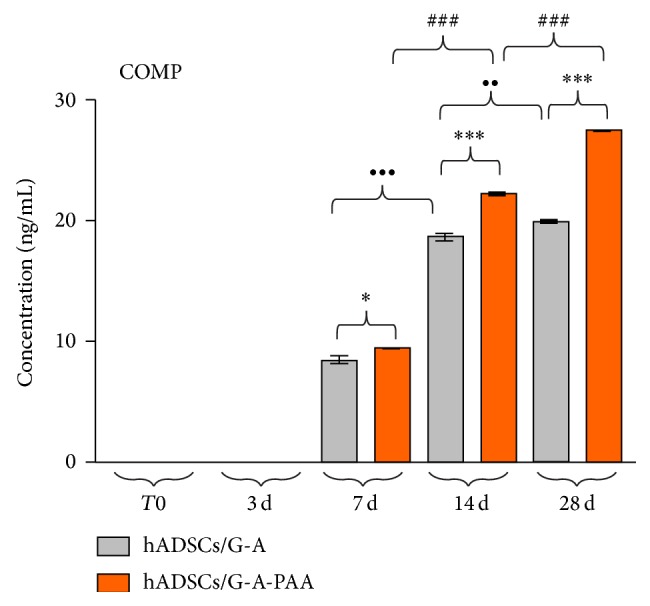
COMP expression in hADSCs/G-A-PAA and hADSCs/G-A bioconstructs exposed to chondrogenic conditions for 28 days, as revealed by ELISA immunoassay (^*^ *P* < 0.05 (hADSCs/G-A-PAA versus hADSCs/G-A: 7 days); ^***^ *P* < 0.001 (hADSCs/G-A-PAA versus hADSCs/G-A: 14 days and 28 days); ^••^ *P* < 0.01 (hADSCs/G-A: 28 days versus 14 days); ^•••^ *P* < 0.001 (hADSCs/G-A: 14 days versus 7 days); ^###^ *P* < 0.001 (hADSCs/G-A-PAA: 14 days versus 7 days and 28 days versus 14 days)).

COMP protein expression was quantitatively evaluated in both bioconstructs using ELISA immunoassay. The results (Figure 9) revealed an increasing trend of COMP protein expression between 7 and 28 days of induced chondrogenic differentiation. The difference (*P* < 0.05) registered in protein levels at 7 days between hADSCs/G-A-PAA and hADSCs/G-A biohybrids increased to a higher significance (*P* < 0.001) during the last two weeks of experiment. COMP expression significantly increased (*P* < 0.001) between 7 and 14 days in the control system and then registered a lower increase (*P* < 0.01) for the last experimental interval, whereas the increase in COMP protein expression in hADSCs/G-A-PAA bioconstruct was found to be statistically significant (*P* < 0.001) and constant in both time intervals.

The data obtained for COMP protein expression are in total accordance with COMP gene expression profile, confirming COMP relation to ECM accumulation and its status as a late chondrogenic marker.

## 4. Discussion

Articular cartilage is a highly specialized tissue, which has a particular function in protecting the bone ends from the forces associated with high mechanical load, thus reducing joint friction. Due to its aneural, avascular, and alymphatic structure, cartilage is a complex and particular type of tissue [24], which possesses a limited self-regeneration potential. Due to these cartilage characteristics, strong clinical requirements have conducted to the development of new strategies for cartilage tissue engineering (CTE).

In an effort to generate cartilage*-like* tissue, a variety of polymers including collagen [25, 26], gelatin [27], silk [28], alginate [29], hyaluronan [30], chitosan [31], agarose [32], polyethylene glycol [33], polyglycolide [34], poly(lactic-*co*-glycolic acid), and hybrids of synthesized or natural materials [35–37] have been tested to date. Polymers' major advantages are the chemical, physical, and functional resemblance with macromolecular constituents of the ECM. Despite appropriate biodegradability and biocompatibility, some macromolecular components do not possess appropriate mechanical properties or biodegradation rate. A combination of different natural and synthetic polymers could in principle provide appropriate biodegradability, biocompatibility, and surface characteristics and mimic the appropriate microenvironment, to support cell adhesion and chondrogenic differentiation [38, 39]. Particularly, a tricomponent IPN based on G, A, and PAA has been recently demonstrated to present tunable properties with respect to water affinity, biodegradability, mechanical behavior, and the capacity to modulate the porosity [22]. It has been shown that through the combination of these three materials, synergistic effects can be obtained. Consequently, such IPNs are ideal for applications in soft TE.

Previous reports have shown that both BM-MSCs and hADSCs provide attractive cell sources for CTE* in vitro* and* in vivo* [40], but hADSCs seem to possess more advantages for current clinical applications. Several studies have shown the potential use of hADSCs in different CTE approaches by demonstrating the chondrogenic differentiation potential of these cells (i) when cultured using the typical aggregate culture technique (pellet culture) [41], (ii) when seeded in materials, or (iii) when implanted in different animal models [42–44]. Awad et al. [45] compared several different scaffolds based on agarose, alginate, and gelatin as support materials for hADSC adhesion, proliferation, and chondrogenic differentiation and concluded that these cells were able to differentiate into chondrocytes when cultured into any of these scaffolds [46].

In our studies we showed that G-A-PAA scaffold is a good candidate for cartilage tissue regeneration purposes due to its good physical, chemical, and structural properties, its biocompatibility, and, not least, its prochondrogenic potential. With respect to the cell distribution, shape, and proliferation trend, valuable information was obtained through a critical comparison of results obtained from complementary techniques, such as SEM and confocal microscopy outcomes (Figure 3). This corroboration also revealed the materials' behavior in plain culture medium during one week of incubation. We noticed that the scaffolds maintained an interconnected porous pattern after 7 days of incubation in plain culture medium (Figure 2(a1)/(b1) and Figure 2 (a4)/(b4)). This pattern allowed further hADSCs efficient penetration in the deeper layers of the structures (Figure 2 (a3)/(b3) and Figure 2 (a6)/(b6)). On the other hand, although negative control PAA scaffold's inner structure was proved to be porous and highly structured (Figure 1(c)), a compact thin polymer layer was noticed on the surface of the hydrogel (Figure 1(d)). This superficial film did not allow cellular infiltration.

Moreover, the tricomponent G-A-PAA scaffold preserved the highly ordered porosity pattern and the well-defined interconnected pores, when compared to the bicomponent G-A control matrix (Figure 2), probably due to the presence of PAA in its formulation. In addition, the quantitative and qualitative biocompatibility investigations performed on both bioconstructs showed that our G-A-PAA engineered system was able to support hADSCs survival, adhesion, and proliferation despite its synthetic PAA component. We concluded that the presence of this polymer offered better properties to G-A-PAA scaffold in terms of structure, stability, pore dimensions, and cell distribution, as compared to the reference matrix.

The actin and tubulin filaments revealed by confocal microscopy (Figure 3) confirmed hADSCs characteristic spindle-*like* morphology in contact with both biomaterials, suggesting cells capacity to adapt to the provided conditions. Even with the presence of PAA synthetic compound in its formulation, G-A-PAA biomatrix displayed a proper microenvironment that allowed hADSCs attachment and cytoskeleton dynamics. Furthermore, 3D reconstructions of laser scanned hADSCs/G-A-PAA and hADSCs/G-A bioconstructs revealed a net positive ratio between living and dead cells inside the biomaterials.

Interestingly, grouped cell proliferation was observed in hADSCs/G-A-PAA system during one week of culture (Figure 2 (a3)/(b3)), probably favored by the three-dimensional porous architecture of the scaffold. Very importantly, at 24 h after seeding hADSCs followed the irregular shape of G-A control matrix (Figure 2 (a5)/(b5)). Unlike G-A-PAA, the bicomponent substrate only promoted proliferation with the formation of a monolayer*-like* distribution (Figure 2 (a6)/(b6)). Considering that these scaffolds were designed for CTE applications and that cell condensation is a critical step in stem cells commitment to chondrogenesis, we concluded that G-A-PAA IPN displays structural advantages over G-A control. Consequently, G-A-PAA biomatrix was used as hADSCs temporary artificial microenvironment for further* in vitro* chondrogenesis studies.

Chondrogenesis is a multistep pathway during which mesenchymal cells first commit to the chondrogenic cell fate, subsequently condense, undergo morphological changes, and turn on cartilage specific genes [47]. Precursor cells condensation is one of the early events in chondrogenesis. The commitment towards the chondrogenic lineage depends on some initiation signals, represented by the cell-cell and cell-matrix interactions and it is associated with changes in the cytoskeleton architecture and an increase in cell adhesion [48]. Sox9 is one of the chondrogenic markers expressed by cells in early stages of condensation. Sox transcription factors were originally identified as an* sry* gene located on chromosome Y. Sox factors contain a high-mobility-group box (HMG) domain, which contributes to its attachment to the small groove of DNA and to the interaction with other transcription factors [49, 50]. Lefebvre et al. [51] and Bell et al. [52] previously demonstrated that Sox9 is a potent activator of Col2a1 gene expression, as it binds to a high-mobility group (HMG) box present in the enhancer of Col2a1 gene, thus strongly promoting transcription of Col2a1 reporter genes. The transcriptional complex includes Sox5 and Sox6, which are also essential for chondrogenic development, but cannot activate Col2a1 gene expression in the absence of Sox9 [53]. Sox9 is essential for cell commitment towards chondrogenic pathway and for the activation of genes encoding chondrocyte-specific matrix proteins, such as collagen type II (Col2a1) [54], aggrecan [55, 56], matrilin-1 [57], and COMP [58]. These proteins are required for maintaining the biochemical properties of articular cartilage.

CEP68 is considered a new marker for the chondrogenic differentiation process, which efficiently complements collagen type II expression in regenerative CTE approaches [59, 60]. CEP68 functions as an ECM protein, which allows discrimination of chondrocytes from osteoblasts and MSCs in cell cultures [60].

COMP, which is predominantly found in the ECM of cartilage, tendons, and ligaments [61], is a member of the Thrombospondin (TSP) calcium-binding protein family. It plays a crucial role in endochondral ossification and in the assembly and stabilization of the ECM, while maintaining the structural integrity of the cartilage through its interaction with aggrecan, fibronectin, and matrilin [62–64]. COMP is able to bind to collagens types I and II via its C-terminal globular domain and thus, it acts as a catalyst to promote fibril formation [65]. Due to its interaction with cell adhesion molecules, COMP was found to favor cellular attachment [66] and also to initiate the transition from chondroprogenitor cells to fully committed chondrocytes, thus enhancing chondrogenesis [48].

In our* in vitro* approach, hADSCs underwent chondrogenic induction by growth factors in both G-A and G-A-PAA scaffolds, demonstrating upregulation of cartilage specific genes and the synthesis of cartilaginous proteins. In our experiment, Sox9 was investigated as an early chondrogenic marker and was found to be expressed at higher levels in hADSCs/G-A-PAA bioconstruct as compared to hADSCs/G-A. This can be interpreted as a more efficient initiation of the chondrogenic program in the hADSCs condensed clusters, which were allowed to form in the structured pores of G-A-PAA scaffold, as previously discussed. Col2a1 synthesis began earlier in the hADSCs/G-A-PAA system than in the reference system. This observation is in accordance to the general findings, which state that collagen type II is an early, very abundant, and highly specific product of differentiating chondrocytes [67]. Under the conditions used in this study, hADSCs revealed an overall greater chondrogenic response when cultivated in the G-A-PAA than in the G-A scaffold, as indicated by higher Col2a1, COMP, and CEP68 upregulation and more extensive matrix synthesis.

**Figure 10:**
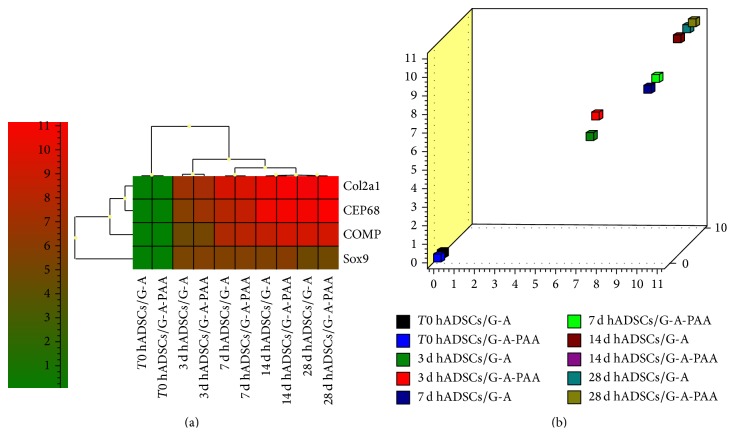
Sox9, Col2a1, CEP68, and COMP chondrogenic markers gene expression analysis via GenEx software generated (a) a heat map and (b) a scatter plot revealing 3D distribution of samples depending on the expression of related ECM markers Col2a1, CEP68, and COMP.

Furthermore, we simultaneously compared chondrogenic marker expression during 28 days of* in vitro* chondrogenesis. Heat map analysis (Figure 10(a)) revealed different patterns of gene expression for the Sox9 chondrogenic inducer, as compared to ECM markers Col2a1, CEP68, and COMP expression. The highest levels of gene expression were registered for Col2a1 and CEP68 at 28 days postchondrogenic induction in both constructs. In contrast, Sox9 was highly and constantly expressed between 3 and 14 days of chondrogenesis but was significantly decreased at day 28 in both culture systems. Thus, the heat map analysis confirmed the distribution of gene expression pattern corresponding to early and late chondrogenic markers. Based on the principle that genes that form a cluster have similar expression pattern, we can predict that in our analysis Col2a1 and CEP68 were the most related genes, together with COMP. This result is in accordance with the findings of Steck et al., 2001 [60], who concluded that CEP68 efficiently complements collagen type II expression in regenerative CTE approaches.

Based on this hierarchical agglomerate clustering, we further analyzed 3D sample distribution for this cluster of cartilage ECM markers and we obtained a graphical representation in the form of a scatter plot (Figure 10(b)). This plot displayed information on sample distribution in groups as a result of the cumulated activity of Col2a1, CEP68, and COMP genes and highlighted the aligned spatial distribution of their expression in hADSCs/G-A and hADSCS/G-A-PAA pairs/time.

## 5. Conclusions

The current study is a proof-of-concept investigation of a new CTE approach to examine the chondrogenic regeneration potential of hADSCs and the advantages of natural polymeric 3D biomaterials. G-A-PAA scaffold proved to better maintain its internal IPN structure and pore shape than G-A control scaffold, probably due to the presence of the synthetic PAA polymer in its formulation. Its porous regulated pattern allowed hADSCs proliferation in groups of clustered cells, which favored chondrogenic condensation, a critical step in chondrogenesis. Despite the presence of PAA in the formulation of our sample, hADSCs were able to equally adhere to both G-A-PAA and G-A substrates, developing a functional cytoskeleton and adopting a characteristic spindle*-like* cell shape. Additionally, G-A-PAA scaffold supported cellular proliferation and viability and showed no cytotoxic effect on hADSCs, confirming its good biocompatibility. Taken together, all these features led us to employ our hADSCs/G-A-PAA bioconstruct for further differentiation studies. Our chondrogenic differentiation approach revealed that G-A-PAA biomaterial ensured an appropriate microenvironment for hADSCs to commit towards the chondrogenic lineage. The chondrogenic markers assessed in our experimental conditions showed higher levels of expression in hADSCs/G-A-PAA as compared to hADSCs/G-A biohybrids. The identification of collagen type II, CEP68, and COMP late chondrogenic markers suggests that we obtained functional chondrocytes from hADSCs. In conclusion, G-A-PAA biomaterial displayed an overall adequate profile for* in vitro* chondrogenesis approaches, which makes hADSCs/G-A-PAA biohybrid an attractive system for prospective* in vivo* CTE applications.

## Figures and Tables

**Table 1:** Primer sequences used to identify early and late chondrogenic markers.

Target	Nucleotide sequence	Fragment length
Sox9 F	5′-TTGAGCCTTAAAACGGTGCT-3′	244 bp
Sox9 R	5′-CTGGTGTTCTGAGAGGCACA-3′	

Col2a1 F	5′-TCACGTACACTGCCCTGAAG-3′	213 bp
Col2a1R	5′-TGCAACGGATTGTGTTGTTT-3′	

CEP68 F	5′-TCTTGTCCCATGGAGAGTCC-3′	154 bp
CEP68 R	5′-GCCCCACTCTTCTTGGTGTA-3′	

COMP F	5′-CCATGGACTTGGCCAGTAGG-3′	385 bp
COMP R	5′-GAGGAAGAGCCAGTGACGAAA-3′	

TBP F	5′-AGGCATCTGTCTTTGCACAC-3′	166 bp
TBP R	5′-GGGTCAGTCCAGTGCCATAA-3′	

YWHAZ F	5′-AGGAGATAAAAAGAACATCCAGTCA-3′	269 bp
YWHAZ R	5′-TATTCTCGAGCCATCTGCTGTTT-3′	
